# Neuroprotective Effects of Cyclosporine in a Porcine Pre-Clinical Trial of Focal Traumatic Brain Injury

**DOI:** 10.1089/neu.2018.5706

**Published:** 2018-12-14

**Authors:** Michael Karlsson, Bryan Pukenas, Sanjeev Chawla, Johannes K. Ehinger, Ross Plyler, Madeline Stolow, Melissa Gabello, Matilda Hugerth, Eskil Elmér, Magnus J. Hansson, Susan Margulies, Todd Kilbaugh

**Affiliations:** ^1^ Department of Anesthesiology and Critical Care Medicine, Children's Hospital of Philadelphia, Perelman School of Medicine, University of Pennsylvania, Philadelphia, Pennsylvania.; ^2^ Mitochondrial Medicine, Department of Clinical Sciences, Lund University, Lund, Sweden.; ^3^ Department of Neurosurgery, Rigshospitalet, Copenhagen, Denmark.; ^4^ NeuroVive Pharmaceutical AB, Lund, Sweden.; ^5^ Department of Radiology, Hospital of the University of Pennsylvania, Perelman School of Medicine, University of Pennsylvania, Philadelphia, Pennsylvania.; ^6^ Department of Bioengineering, University of Pennsylvania, Philadelphia, Pennsylvania.

**Keywords:** magnetic resonance imaging, magnetic resonance spectroscopy imaging, mitochondria, traumatic brain injury

## Abstract

Mitochondrial dysfunction is thought to be a hallmark of traumatic brain injury (TBI) and plays a pivotal role in the resulting cellular injury. Cyclophilin D–mediated activation of the mitochondrial permeability transition pore has been suggested to contribute to this secondary injury cascade. Cyclosporine possesses neuroprotective properties that have been attributed to the desensitization of mitochondrial permeability transition pore activation. *In vivo* animal experiments have demonstrated neuroprotective effects of cyclosporine in more than 20 independent experimental studies in a multitude of different experimental models. However, the majority of these studies have been carried out in rodents. The aim of the present study was to evaluate the efficacy of a novel and cremophor/kolliphor EL–free lipid emulsion formulation of cyclosporine in a translational large animal model of TBI. A mild-to-moderate focal contusion injury was induced in piglets using a controlled cortical impact device. After initial step-wise analyses of pharmacokinetics and comparing with exposure of cyclosporine in clinical TBI trials, a 5-day dosing regimen with continuous intravenous cyclosporine infusion (20 mg/kg/day) was evaluated in a randomized and blinded placebo-controlled setting. Cyclosporine reduced the volume of parenchymal injury by 35%, as well as improved markers of neuronal injury, as measured with magnetic resonance spectroscopic imaging. Further, a consistent trend toward positive improvements in brain metabolism and mitochondrial function was observed in the pericontusional tissue. In this study, we have demonstrated efficacy using a novel cyclosporine formulation in clinically relevant and translatable outcome metrics in a large animal model of focal TBI.

## Introduction

Traumatic brain injury (TBI) is caused by physical trauma to the head or a rapid acceleration-deceleration. TBI is a leading cause of death and disability, and in the United States alone, 2.8 million people sustain a TBI each year, resulting in nearly 50,000 deaths.^1^ Those that survive may not only suffer from long-term physical disabilities, but also cognitive disorders, including depression, drug and alcohol abuse, and increased risk of suicide.^2,3^ Despite the enormous medical need, there is currently no approved neuroprotective treatment for TBI.

After the primary injury, associated with immediate structural damage and potential loss of brain tissue, there is a subsequent secondary injury that continues to evolve for days after the primary insult. Mitochondrial dysfunction and oxidative stress are thought to play a pivotal role in this secondary injury cascade.^4^ Specifically, the opening of the mitochondrial permeability transition pore (mPTP), as a result of excitotoxicity and calcium overload, has been proposed to be a decisive pathophysiological mechanism of the secondary injury.^5–8^ The opening of the mPTP leads to loss of mitochondrial inner membrane integrity and adenosine triphosphate (ATP) production, generation of reactive oxygen species (ROS), and release of proapoptotic factors.^9–12^ The composition of the mPTP is debated, but a key regulatory component of the mPTP is cyclophilin D (CypD).

Cyclosporine is a drug in clinical use for immunosuppression in transplant medicine, marketed as Sandimmune^®^. In addition to its immunosuppressive effects, it was also discovered to possess neuroprotective effects through inhibition of the CypD-dependent and calcium-mediated activation of the mPTP.^7,8,13–18^ Cyclosporine also prevents excess formation of ROS through inhibition of mPTP, further preventing oxidative damage.^19–22^ More than 20 independent experimental *in vivo* studies, in a multitude of TBI models with various outcome metrics, have demonstrated neuroprotective effects of cyclosporine.^12,23–44^ Further, two clinical studies with cyclosporine in TBI have also been completed, demonstrating safety in this patient population, as well as indicating a positive treatment effect.^45–47^ There are, however, a few studies that have failed to show efficacy with cyclosporine, primarily when screening multiple drugs in a high-throughput study design.^48,49^

The majority of the pre-clinical efficacy studies have been performed in rodent models and utilized endpoints that are not directly translatable to humans. First, though critical for obtaining mechanistic understanding, rodents may have different bioenergetic response after brain injury from gyrencephalic animals. Second, the size of the pig brain allows for better neuroimaging of cortical and subcortical structures for assessment of injury. Rodents also have a significantly higher gray-to-white-matter ratio (GWR), which limits scaling, and it is therefore difficult to use outcome metrics related to the characterization of the diffuse axonal injury.^50^ Third, the gyrencephalic pig brain is more similar to the human brain in regard to neuroanatomy, GWR, and development compared to rodents.^31,51–53^

The overall aim of this study was to evaluate the efficacy of a novel cyclosporine formulation in a large animal model of focal TBI using a translational study design. NeuroSTAT^®^ is a novel lipid emulsion containing cyclosporine that differs from the Sandimmune formulation, which contains the solubilizer, Kolliphor EL (previously named Cremophor EL), which is known to cause hypersensitivity reactions in some patients, ranging from skin reactions to potentially fatal anaphylactic shock.^54^ Further, a secondary aim was to perform a systematic pharmacokinetics (PK) evaluation in order to create a bridge between the existing pre-clinical studies, primarily in rodents, and human clinical trials for TBI.

## Methods

### Outline of study design

#### Step 1: Comparative pharmacokinetic trial

As a first step, we performed a 24-h comparative PK trial of the NeuroSTAT and Sandimmune intravenous (i.v.) formulations of cyclosporine in a large animal model of focal controlled cortical impact (CCI) injury to compare PK profiles of both cyclosporine formulations in swine. We have previously shown bioequivalence between cyclosporine formulations in humans.^54^ Piglets were randomized to 20 mg/kg/day (i.v.) of either the NeuroSTAT or Sandimmune formulation of cyclosporine (*N* = 3/group). One hour post-TBI, a bolus dose was i.v. administered, with bolus/infusion ratio of 0.3 administered over 5 min, followed by a continuous infusion for 24 h.

#### Step 2: Dose escalation

We then performed a 24-h PK dose escalation study using the NeuroSTAT formulation in the same model to determine optimal dosing for the efficacy study. Optimal dosing was determined based on blood exposure as well as brain concentrations as compared to the previous literature, including the clinical studies, with minimal evidence of toxicity. In addition to the 20 mg/kg/day dose evaluated in step 1, we also evaluated 5, 10, and 40 mg/kg/day of NeuroSTAT (*N* = 3/group), administered as described above.

#### Step 3: Efficacy trial

**FIG. 1.**
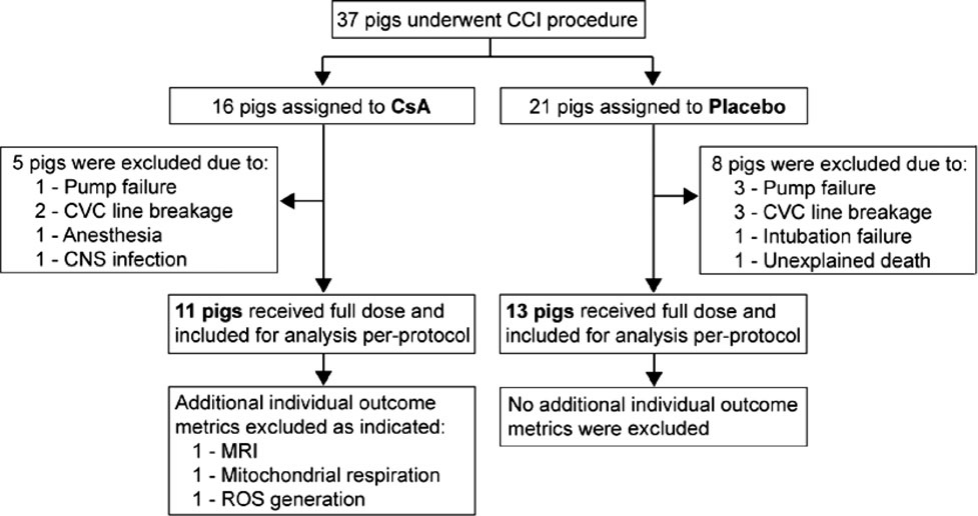
Randomization and inclusion for analysis per protocol. The per-protocol population includes all the pigs that underwent randomization and received the full dose. All data underwent quality control (QC), and all exclusions of data points were performed before unblinding. CCI, controlled cortical injury; CsA, cyclosporine; CVC, central venous catheter; CNS, central nervous system; MRI, magnetic resonance imaging; ROS, reactive oxygen species.

Informed by data from steps 1 and 2, we then evaluated the NeuroSTAT formulation of cyclosporine at the dose of 20 mg/kg/day, in a 5-day, randomized, blinded, placebo-controlled efficacy study. The bolus dose was administered as described above, and the infusion was continued for 5 days at 20 mg/kg/day. A total of 37 animals underwent randomization, of which 24 received the full dose (*n* = 11 cyclosporine and *n* = 13 placebo) and were subsequently included for analysis of outcome metrics as indicated in Figure 1.

### Animals

The study was carried out in accord with the recommendations in the Guide for the Care and Use of Laboratory Animals of the National Institutes of Health (NIH). All procedures were approved by the Institutional Animal Care and Use Committee of the University of Pennsylvania (Number: 803401). Four-week-old (7–9 kg) Yorkshire piglets, with brain development and characteristics similar to human toddlers, were studied. Based on previous work, females were utilized to limit heterogeneity.

Fasted piglets were pre-medicated with an intramuscular injection of ketamine (20 mg/kg) and xylazine (2 mg/kg). Subjects were intubated after induction with 4% inhaled isoflurane using a 1.0 fraction of inspired oxygen by snout mask, until abolishment of response to an interdigital hoof pinch. Anesthesia was maintained with approximately 1% inhaled isoflurane by endotracheal tube with fraction of inspired oxygen set to 0.21. Anesthetic depth was characterized by a relaxed jaw tone, absence of foot withdrawal in response to a firm interdigital pinch, and absence of palpebral and corneal reflexes. If any of these signs were absent, depth of anesthesia was altered by increasing the percentage of isoflurane. Preceding injury, buprenorphine SR (0.1 mg/kg) was also delivered intramuscularly for analgesia, and Cefazolin (30 mg/kg) was injected intramuscularly as a prophylactic antibiotic. A circulating water blanket kept core body temperature constant between 36 and 38°C monitored by a rectal probe. Throughout the experiment, blood pressure, oxygen saturation, respiratory rate, heart rate, and end-tidal CO_2_ were continuously monitored (VetCap model 2050081; SDI, Waukesha, WI). Mechanical ventilation, if necessary, was adjusted to maintain normoxia and normocarbia, titrated to peripheral saturation of greater than 94% and an end-tidal CO_2_ level of 35–45 mm Hg.

### Placement of central venous catheters

Before injury, two tunneled central venous catheters (CVCs) were placed into bilateral cephalic veins, with termination in the superior vena cava and right atrial junction. The lines were tunneled between the animals' scapula. One CVC line was connected to an ambulatory Bluetooth pump (3D BT mini infusion; Strategic Applications Inc., Infusion Technologies, Lake Villa, IL) for continuous drug delivery, whereas the other CVC was used for serial blood collection.

### Controlled cortical impact injury

A mild-to-moderate focal contusion injury (corresponding to clinical human severity classifications) was induced using a spring-loaded CCI device with a typical lesion volume of 8% of the hemisphere or 4% of the cerebrum as previously described.^55^ In short, while maintained on anesthesia, the head was trimmed and prepped with chlorhexidine solution. The right coronal suture was exposed, a craniectomy was performed, and the exposed dura was opened to reveal the cortical surface. The device was stabilized against the skull with screws and the spring-loaded tip rapidly (4 ms) indented to a depth of 0.7 cm of the cortical rostral gyrus. The device was removed and the dura reapproximated. Finally, the surgical flap was sutured closed. After emergence from anesthesia, the piglets were extubated. Animals were continuously monitored during recovery. They all displayed initial depressed activity and gait instability, but neither hypotension nor desaturation was ever observed after injury in any subject. Subjects were returned to the animal housing facility when they met the following criteria: vocalization without squealing, able to ambulate, devoid of aggression or avoidance behavior, absence of piloerection, and unassisted feeding and drinking.

### Sample acquisition

Before sacrifice the animals were reanesthetized as described above. While under general anesthesia, a 24-gauge spirotte spinal needle was placed at lumbar level into the intrathecal space for removal of approximately 23 mL of cerebrospinal fluid (CSF). A wide bilateral craniectomy was subsequently performed and the brain was rapidly extracted while simultaneously receiving a pentobarbital (150 mg/kg i.v.) overdose. For the PK studies, heart, liver, and kidney samples were harvested immediately after sacrifice, and immediately frozen at −80°C.

### Pharmacokinetics

Analysis of cyclosporine levels was carried out using liquid chromatography/tandem mass spectrometry by a contract research organization (Alliance Pharma, Devault, PA).

### Neuroimaging

In the efficacy study, the animals were anesthetized again on day 5 as described above, and magnetic resonance imaging (MRI) and magnetic resonance spectroscopic imaging (MRSI) sequencing was obtained on a Siemens 3T TRIO MRI research magnet (Seimens, Munich, Germany) using a standard knee coil. Anatomical images (T1-weighted, T2-weighted, fluid-attenuated inversion recovery [FLAIR] weighted, susceptibility-weighted, and diffusion-weighted) were assessed for presence of hemorrhage, edema, and other structural abnormalities. MRI criteria for injury were defined as an area of increased signal abnormality on FLAIR imaging relative to the contralateral (uninjured) hemisphere. Images were analyzed using TeraRecon v4.4.12.194 (TeraRecon, Foster City, CA) software and interpreted by a board-certified neuroradiologist blinded to treatment group. For each subject, the area of injury on each slice was manually traced and volume of injury was then automatically calculated by the software.

Single-slice two-dimensional multi-voxel ^1^H MRSI was performed using a spin echo (point resolved spectroscopy) sequence with water suppression by means of a chemical shift selective saturation pulse. Sequence parameters included: repetition time/echo time (TE) = 1700/30 ms, number of excitations = 3, field of view = 16 × 16 cm^2^, matrix size = 16 × 16, slice thickness = 20 mm resulting in a voxel size of 10 × 10 × 20 mm^3^, bandwidth = 1200 Hz, flip angle = 90 degrees, and vector size = 1024. Volume of interest (VOI) was selected so as to include regions of injury and normal brain parenchyma avoiding the scalp, skull base, or sinuses. Outer volume saturation slabs (30 mm thick) were placed outside the VOI to suppress lipid signals from the scalp. The data set was acquired using elliptical k-space sampling with weighted phase encoding to reduce the acquisition time. Manual shimming was performed to achieve an optimal full width half maximum of <20 Hz (magnitude spectrum) of the water signal. A water unsuppressed ^1^H MRSI spectrum was also acquired to use the water signal for computing metabolite concentrations.

Absolute concentrations of metabolites were measured using a user-independent spectral fit program (linear combination [LC] model).^56,57^ The region between 0.2 and 4.0 parts per million of the spectrum was analyzed and the following metabolites were evaluated: N-acetylaspartate (NAA), gamma-aminobutyric acid (GABA), phosphocreatine (PCr), taurine (Tau), creatine (Cr), choline (GPC and PCh), myoinositol (Ins), Glx (glutamate + glutamine), lactate (Lac), glutathione (GSH), alanine (Ala), and aspartate (Asp). The error in the spectral fitting routine (LC model) was used to assess the spectral quality for a particular voxel; metabolite concentrations from only those voxels were used that had Cramer-Rao lower bounds/standard deviations (SDs) of less than 20% for all the metabolites. Short TE (TE30) ^1^H-MRS metabolites NAA, GABA, PCr, Tau, Cr, GPC and PCh, Ins, Glx, Lac, GSH, Ala, and Asp were measured and quantified using the LC model.

### Cerebral microdialysis

After neuroimaging, cerebral microdialysis was performed in the ipsilateral hemisphere 10 mm from the contusion (CMA 71 Elite; M Dialysis AB, Stockholm, Sweden). While under anesthesia, as described above, the scalp was reopened and the microdialysis probe was placed in the brain parenchyma after needle puncture of the dura mater. Probes were placed 1 cm deep to reach the junction of cortex and subcortical white matter. Sterile saline was perfused at 1 μL/min, and after a 30-min equilibration period, samples were collected over the succeeding 30 min. Samples were labeled and immediately frozen at −80°C and subsequently analyzed by ISCUS Flex™ Microdialysis Analyzer (M Dialysis AB) by a blinded technician.

### Mitochondrial high-resolution respirometry

After neuroimaging and collection of cerebral microdialysis, tissue was harvested as described above. A 2 cm^2^ region of cortex of visibly viable tissue was resected immediately adjacent to the rostral edge of the contusion, along with a corresponding mirrored region from the contralateral hemisphere and placed in ice-cold buffer (320 mM of sucrose, 10 mM of Trizma base, and 2 mM of ethylene glycol tetraacetic acid [EGTA]). Adherent necrotic tissue from the contused area was dissected and disposed. In addition, subcortical white matter and blood vessels were removed through dissection. Tissue was subsequently quickly placed on drying paper to absorb excess buffer and weighed. Tissue was then gently homogenized on ice in MiR05 (110 mM of sucrose, 0.5 mM of EGTA, 3.0 mM of MgCl_2_, 60 mM of K-lactobionate, 10 mM of KH_2_PO_4_, 20 mM of taurine, 20 mM of HEPES, and 1.0 g/L of fatty acid–free bovine serum albumin) using a 5-mL Potter–Elvehjem Teflon and glass homogenizer to a concentration of 1 mg of wet weight tissue/10 μL of MiR05 buffer.

Mitochondrial respiratory function was analyzed *ex vivo* in brain cortex homogenates using high-resolution respirometry (Oxygraph-2k; Oroboros Instruments, Innsbruck, Austria) with a substrate-uncoupler-inhibitor titration (SUIT) protocol with sequential additions, as previously described.^55^ In the utilized SUIT protocol, oxidative phosphorylation capacities with electron flow through both complex I (CI) and complex II (CII) were evaluated as well as the convergent electron input through the Q-junction (CI + CII) using the nicotinamide adenine dinucleotide–linked substrates, malate (5 mM) and pyruvate (5 mM), and succinate (10 mM), both in the presence of adenosine diphosphate (1 mM), corresponding to state 3 respiration in established bioenergetics terminology.^55^ Oligomycin, an inhibitor of the ATP synthase, induced mitochondrial respiration independent of ATP production across the inner mitochondrial membrane, referred to as LEAK respiration (LEAK_CI + CII_) or State 4_O_. Maximal convergent nonphosphorylating respiration of the electron transport chain (ETC_CI +_ _CII_) was evaluated by titrating the protonophore, carbonyl cyanide p-(trifluoromethoxy) phenylhydrazone. Respiration exclusively through complex II (ETS_CII_) was achieved through the addition of rotenone (2 mM). The complex III inhibitor, antimycin-A (1 μg/mL), was added to measure the residual oxygen consumption (ROX) that is independent of the ETC, and this was subtracted from each of the measured respiratory states. Complex IV activity was measured by the addition of N,N,N′,N′-tetramethyl-pphenylenediamine (TMPD; 0.5 mM) together with ascorbate (ASC; 0.8 mM). The complex IV inhibitor, sodium azide (10 mM), was added to reveal the remaining chemical background that is subtracted from the TMPD-induced oxygen consumption rate.

### Fluorometry and measurement of reactive oxygen species

Measurements of ROS generation were assessed simultaneously with respirometry measurements by a O2k-Fluorescence LED2-Module (Oxygraph-2k; Oroboros Instruments), utilizing an Amplex UltraRed assay, as previously described. In short, in the presence of horseradish peroxidase (1 U/mL), Amplex UltraRed (N-acetyl-3,7 dihydroxyphenoxazine) (5 mM) reacts with H_2_O_2_ to produce the fluorescent compound, resorufin. The addition of superoxide dismutase (10 U/mL) ensures that all superoxide is converted into H_2_O_2_.

### Citrate synthase activity

Citrate synthase (CS) activity was measured as a marker of mitochondrial content. Chamber contents from mitochondrial high-resolution respirometry measurements were frozen for subsequent CS activity measurements. A commercially available kit (Citrate Synthase Assay Kit, CS0720; Sigma-Aldrich, St. Louis, MO) was used, according to the manufacturer's instructions, to determine CS activity (μmol/mL/min).

### Statistical analysis

Data reporting was based upon the National Institute of Neurological Disorders and Stroke Common Data Elements (CDE) for pre-clinical TBI research.^58^ CDE for individual animals will be available in the Federal Interagency Traumatic Brain Injury Research informatics system. In steps 2 and 3, animals were randomly assigned to one of the treatment groups by a study monitor not performing the experiment or analyses, in permuted blocks of 4. Drug and placebo vehicle were individually coded and identical vials were utilized. All participants performing the study and analyzing outcome metrics were completely blinded to treatment group. The randomization key was kept in an encrypted folder only accessible by the study monitor.

No interim evaluation of data was carried out. All data underwent quality control before unblinding, and all exclusions of data points were performed at this stage. Data were excluded because of clear laboratory error and using the Grubbs test with α set at 0.01.^59^ One imaging sequence was also excluded because of a technical mistake with an incorrect sequence. The primary and secondary end-points, as outlined in the Results section, were determined before unblinding. An unpaired Student's *t*-test or Mann–Whitney *U* test were used for interval data. For ordinal data, Fisher's exact test was used for two categories and chi-square for three or more categories. A *p* value <0.05 was considered statistically significant. Statistical evaluation was performed using SPSS 23 and Prism 7 (GraphPad Software, San Diego, CA). Results are reported as mean ± standard error of the mean (SEM), except when noted.

## Results

### Pharmacokinetics

**FIG. 2.**
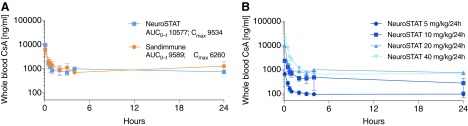
(**A**) Cyclosporine (CsA) concentrations in whole blood. Intravenous dose of NeuroSTAT and Sandimmune (20 mg/kg/24 h). Bolus given 1 h post-injury followed by 24-h infusion. Mean ± SD. (**B**) CsA concentrations in whole blood. Intravenous dose of NeuroSTAT (5–40 mg/kg/24 h). Bolus given 1 h post-injury followed by 24-h infusion. Analysis of cyclosporine levels was carried out using liquid chromatography/tandem mass spectrometry. Mean ± SD. *N* = 3/group. AUC, area under the curve; SD, standard deviation.

**FIG. 3.**
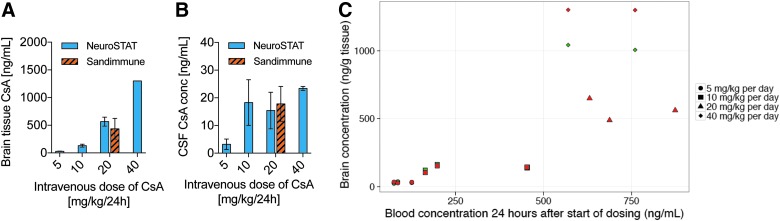
Brain tissue (**A**) and CSF concentrations (**B**) of cyclosporine. Intravenous dose of NeuroSTAT (5–40 mg/kg/24 h) and Sandimmune (20 mg/kg/24 h). Bolus given 1 h post-injury followed by 24-h infusion with subsequent sample collection. Analysis of cyclosporine levels was carried out using liquid chromatography/tandem mass spectrometry. Mean ± SD. (**C**) Individual brain concentrations versus blood concentrations in piglets. Each point represents the data for 1 subject, and the points are colored based on sample location: red = ipsilateral; green = contralateral. Only ipsilateral samples were collected in the 20-mg/kg dose group. *N* = 3/group. CsA, cyclosporine; CSF, cerebrospinal fluid; SD, standard deviation.

PK data are outlined in Table 1. Blood concentrations decreased rapidly after the administration of the i.v. bolus dose, and steady-state concentrations were obtained by the constant infusion. Stable concentrations were obtained by 3–6 h after initiation of treatment until animals were terminated at 24 h (Fig. 2A,B). The comparative PK trial revealed that mean cyclosporine brain tissue concentrations of Sandimmune (20 mg/kg/day) and NeuroSTAT (20 mg/kg/day; 436 ± 187 vs. 566 ± 80 ng/g; *p* = 0.354; Fig. 3A) indicated equivalent brain exposure. Peripheral organ penetrance (heart, liver, and kidney) was also similar between formulations (data not shown). Brain concentrations increased with increasing dose, and, when normalized by dose, the 20- and 40-mg/kg dose groups had very similar cyclosporine concentrations in the brain (Fig. 3A). However, the brain/blood concentration ratio appeared to increase with increasing dose in piglets, which partly may be caused by the deviation from dose proportionality in the blood concentrations (Table 1). Cyclosporine brain concentrations increased with each escalation from 5 to 40 mg/kg/day, indicating increasing blood–brain barrier penetration, but the absolute levels in CSF did not seem to correlate with brain concentration (Fig. 3B). Figure 3C illustrates the individual brain concentrations versus blood concentrations. Cyclosporine delivery into the ipsi- and contralateral hemisphere, relative to injury, was similar for all doses tested. Cyclosporine delivery to brain, both ipsi- and contralateral, was dose dependent with total area under the curve consistent with dosing effects. Renal concentrations did not display a significant difference between 5-, 10-, and 20-mg/kg/day dose; however, the 40-mg/kg dose was significantly higher than all other doses. Two animals had unexpected demise after initiation of 40-mg/kg bolus dose of cyclosporine with death immediately following bolus dose or during administration of bolus dose. Necropsies performed by the attending veterinarian were inconclusive for cause of death. Based on pre-clinical study tolerability, blood exposure, and brain concentrations at 20 mg/kg/day in the pig that were consistent with those measured in previous clinical trials at 5 mg/kg/day (461 ± 118 ng/mL^45^ and 500–600 ng/mL^47^), the dose of 20 mg/kg/day was determined to be optimal and was therefore utilized in the randomized, blinded, placebo-controlled study.

**Table 1.** Mean Blood Concentrations (ng/mL) at Steady State, Mean Brain Concentrations, Mean Ratio of Brain/Blood Concentrations, the Mean of Dose Normalized Blood Concentrations, and Mean of Dose Normalized Brain Concentrations

	*Mean concentration or ratio (SD)*^a^			*Mean concentration/dose*^b^	
*Dose (mg/kg/day)*	*Blood*	*Brain*	*Brain/blood ratio*	*Blood*	*Brain*
5	95.8 (26.6)	30.4 (1.40)	0.331 (0.0747)	19.17	6.08
10	272 (158)	136 (23.3)	0.594 (0.256)	27.17	13.6
20	731 (127)	566 (80.4)	0.793 (0.206)	36.55	28.29
40	665 (134)	1160 (13.4)	1.78 (0.380)	16.63	29.04

^a^ For brain concentration, it is the mean of each piglet's average brain concentrations (right and left brain sample, when applicable).

^b^ Mean concentration/daily dose in mg.

SD, standard deviation.

### Cerebral microdialysis

**FIG. 4.**
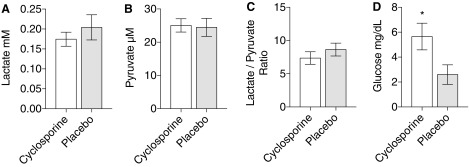
Cerebral microdialysis in the ipsilateral hemisphere 10 mm from the contusion, collected over 1 h, on day 5 post-injury. (**A**) Lactate. (**B**) Pyruvate. (**C**) Lactate/pyruvate ratio. (**D**) Glucose. *Unpaired *t*-test (*p* = 0.028). Mean ± SEM. *N* = 11 (CsA), *N* = 13 (placebo). CsA, cyclosporine; SEM, standard error of the mean.

As demonstrated in Figure 4, there was a decrease in lactate/pyruvate ratio in the cyclosporine group compared to placebo, primarily driven by an increase in lactate in the placebo group. These differences were not statistically significant. Pericontusional cortical glucose levels were significantly increased in the cyclosporine group (5.653 ± 1.068 vs. 2.601 ± 0.7831; *p* = 0.028; Fig. 4D) presented as mg/dL. Presented as mmol/L, this corresponds to 0.314 ± 0.059 versus 0.144 ± 0.043 (*N* = 11 cyclosporine A [CsA] and *N* = 13 [placebo]).

### Mitochondrial respiration

**FIG. 5.**
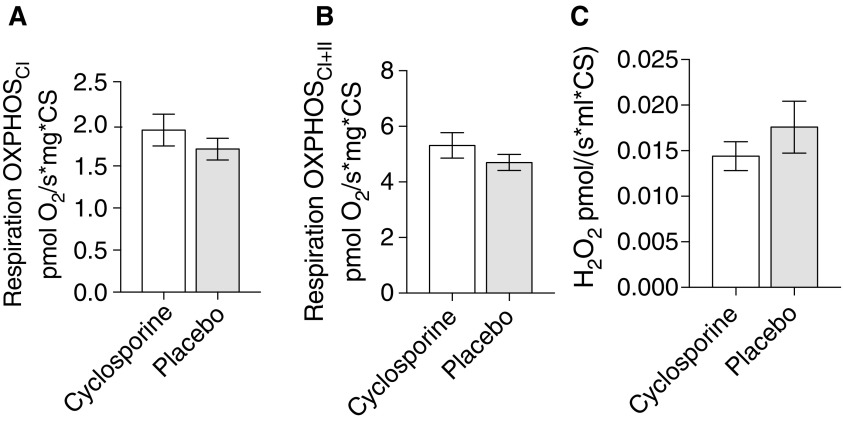
Mitochondrial respiration in brain tissue homogenates collected from the ipsilateral hemisphere immediately adjacent to the rostral edge of the contusion. (**A**) Oxidative phosphorylation (OXPHOS) with complex I substrates malate and pyruvate. (**B**) Maximum oxidative phosphorylation with the addition of succinate. (**C**) Reactive oxygen species (ROS) generation utilizing an Amplex UltraRed assay measuring H_2_O_2_. The addition of superoxide dismutase (SOD) ensures that all superoxide is converted into H_2_O_2_. Measurement with complex I substrates malate and pyruvate. Mean ± SEM. *N* = 10 (CsA), *N* = 13 (placebo). CsA, cyclosporine; SEM, standard error of the mean.

Analysis revealed no statistical difference in respiration between treatment groups, for the pre-defined measures of mitochondrial respiration (OXPHOS_CI_, OXPHOS_CI+CII_, LEAK_CI+CII_, and respiratory control ratio (RCR; OXPHOS_CI+CII_/ LEAK_CI+CII_). However, there was an overall improvement in mitochondrial metrics compared to placebo, supporting a beneficial effect of cyclosporine treatment, with higher OXPHOS (Fig. 5A,B) and lower LEAK, resulting in a favorable RCR (*N* = 10 [CsA] and *N* = 13 [placebo]).

### Reactive oxygen species production and oxidative damage

Analysis revealed no statistical difference in ROS generation between treatment groups for the pre-defined measures corresponding to the respiratory states (OXPHOS_CI_, OXPHOS_CI + CII_, LEAK_CI + CII_). The overall trend displayed was consistently lower ROS generation in the cyclosporine-treated group (Fig. 5C; N = 10 [CsA] and N = 13 [placebo]).

### Neuroimaging

**FIG. 6.**
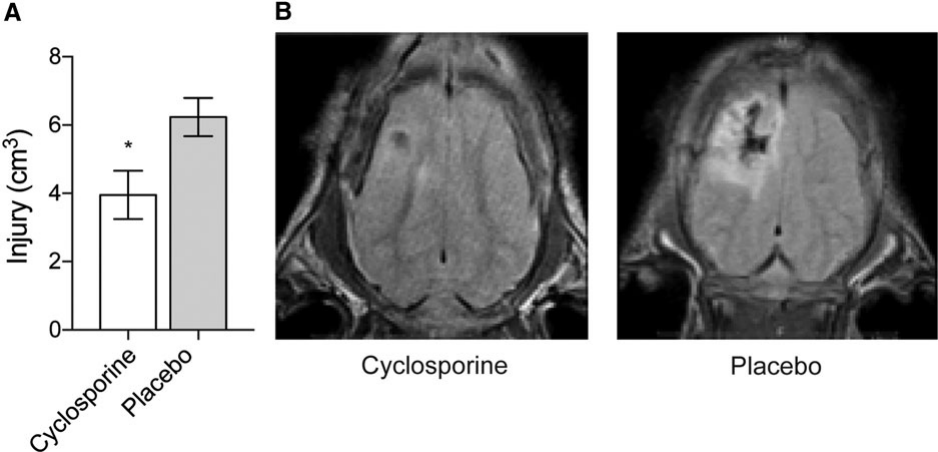
(**A**) Volume of injury (VOI) on MRI measured on day 5 post-injury. VOI was measured and calculated by a blinded neuroradiologist. *Unpaired *t*-test (*p* = 0.018). Mean ± SEM. (**B**) MRI pictures are representative of the median injury in the treated group (left) and the placebo group (right). *N* = 10 (CsA), *N* = 13 (placebo). CsA, cyclosporine; MRI, magnetic resonance imaging; SEM, standard error of the mean.

**FIG. 7.**
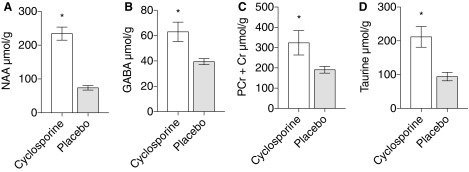
^1^H-MRS metabolites measured on day 5 post injury. Volume of injury was measured and calculated by a blinded neuroradiologist. **(A)** N-acetylaspartate (NAA) (234,1 ± 19,27 vs. 73,48 ± 6,787; *p*-value <0.0001). **(B)** Gamma-aminobutyric acid (GABA) (62,97 ± 7,645 vs. 39,45 ± 2,336; *p*-value = 0.0019). **(C)** Phosphocreatine and creatine (PCr + Cr) (323,5 ± 59,91 vs. 191 ± 16,74; *p*-value = 0.033). **(D)** Taurine (211,7 ± 30,42 vs. 94,33 ± 12,57; *p*-value <0.001). N = 10 (CsA), N = 13 (placebo).

Volume of injury in cortical and subcortical tissue was reduced by 35% compared to placebo, measured with MRI on day 5 (3.955 cm^3^ ± 0.7099 vs. 6.235 cm^3^ ± 0.5581; *p* = 0.018; Fig. 6). ^1^H-MRS metabolites measured 5 days post-TBI displayed significantly higher mean NAA (234 ± 19.27 vs. 73.48 ± 6.787; *p* < 0.0001), GABA (62.97 ± 7.645 vs. 39.45 ± 2.336; *p* < 0.0019), PCr + Cr (323.5 ± 59.91 vs. 191 ± 16.74; *p* = 0.033), and Tau (211.7 ± 30.42 vs. 94.33 ± 12.57; *p* < 0.001) concentrations in treated subjects compared to placebo (Fig. 7). There was no significant difference between groups in other metabolites measured at 5 days post-TBI: GPC and PCh, Ins, Glx, Lac, GSH, Ala, and Asp (*N* = 10 [CsA] and *N* = 13 [placebo]).

## Discussion

In this study of focal TBI, continuous infusion of cyclosporine (20 mg/kg/day) for 5 days significantly reduced volume of parenchymal injury by 35%, as measured by MRI. Additionally, animals treated with cyclosporine displayed increased neuronal viability (NAA), increased inhibitory neurotransmitters (GABA), increased high-energy phosphate species reflecting cellular energetics (PCr), and increased amino acids (Tau) measured by *in vivo* ^1^H-MRS in “at-risk” pericontusional cortical tissue. By day 5 of injury, without ongoing ischemia or a second injury, large alterations in cerebral metabolism with changes in lactate/pyruvate levels, antioxidant defense (GSH), pathological ROS generation, and glutamate and glutamine excitatory neurotransmitter pools is less likely to have shown a significant difference between treatment and placebo. However, a consistent trend toward improvements in brain metabolism and mitochondrial respiratory function, as well as decreased generation of ROS, was still observed with cyclosporine treatment in our molecular analysis. These trends, coupled with a significant improvement in volume of injury on MRI (primary outcome) and decreased neuronal injury on MRSI, lead us to conclude that this was a positive pre-clinical trial with cyclosporine having a significant beneficial effect.

The brain needs unimpaired oxidative phosphorylation for its energy supply, and it has been argued that mitochondrial dysfunction, with a subsequent energy deficit, precedes and perpetuates secondary cell death post-TBI.^4^ As described in the Introduction, cyclosporine binds to CypD in the mitochondrial matrix and consequently inhibits mPTP formation and thereby maintains mitochondrial membrane potential and ATP generation.^60–62^ The ability of cyclosporine to inhibit mPTP formation is well described mechanistically and has been shown in mitochondria isolated from rodent brain, as well as mitochondria isolated from human brain tissue. The previous mechanistic data, together with present and previous efficacy data demonstrating the neuroprotective effects of cyclosporine, support the notion that mitochondrial dysfunction is an important aspect of the secondary injury cascade post-TBI.^7,8,16,63–65^ To simulate a clinical trial as closely as possible, we did not include sham animals in this blinded, randomized, placebo-controlled pre-clinical trial. However, comparing to historical data and recent sham data collected in the 4-week-old swine, there seems to be a persistent decrease in complex I respiration (OXPHOS_CI_ 3.1–4.5 pmol O_2_/mg*s/CS) and convergent oxidative phosphorylation through complexes I and II (OXPHOS_CI + CII_: 6.0–8.5 pmol O_2_/mg*s/CS) at 5 days post-CCI.^66^ Microdialysis lactate/pyruvate ratios in sham animals (7.4 ± 0.93) are similar to injured animals at 5 days (data not shown).

We selected the initial dose for PK on previous published rodent data, our own previous data in the piglet, as well as clinical data using the NeuroSTAT formulation.^24,26,31–33,54^ For the dose-escalation study, we wanted to cover a broad dosage spectrum within the therapeutic range based on the previous literature, to measure a full PK profile and explore dose proportionality. The half-life of cyclosporine is around 18 h in piglets and 7 h in adult pigs.^67^ We utilized a rapid bolus dose to achieve therapeutic concentrations quickly. At the highest dose group during the PK evaluations, 40 mg/kg/day, 2 animals experienced unexpected demise after initiation of the post-TBI bolus dose. The rapid bolus in the present study consisted of 30% of the total daily dose given during the first 5 min of drug treatment. This design was consistent with previous experience from the investigators, but differs compared to how the bolus dose of the investigational drug has been given in clinical studies and what is within the approved label for Sandimmune.^31,32,68,69^ The bolus dose in the 40-mg/kg/day cohort corresponded to 12 mg/kg, administered over 5 min. In the clinical studies, cyclosporine in lipid emulsion has been given as an initial bolus 2.5 mg/kg over 10–15 min. The rate of the bolus infusion in the animals in the highest dosing cohort was thus 10–14 times faster, and the total dose during the whole bolus administration around 5 times higher than what has been used clinically. The rapid bolus in the 40-mg/kg/day cohort of the current study may reflect the highest tolerable dosing rate in the current study setting.

Most previous studies demonstrating efficacy of cyclosporine have utilized the Sandimmune cyclosporine formulation. There have been case reports of serious adverse effects after administration of Sandimmune, as well as when Kolliphor EL has been used as a carrier for other i.v. drugs.^70–76^ Several drugs that previously contained Kolliphor EL as a carrier medium, such as propofol, are now only available as lipid emulsions.^74,77^ The Operation Brain Trauma Therapy consortium failed to show efficacy in their rodent models using the Sandimmune cyclosporine formulation, and they suggested that alternative pharmacotherapies should be tested using cyclosporine preparations where the vehicle does not contain Kolliphor EL given that toxicity was also observed in the vehicle group.^49^

A myriad of therapeutics for TBI have been tested in clinical trials, but, unfortunately, all attempts to find an effective neuroprotective treatment have failed. Most recently, progesterone failed in two large phase III trials.^78,79^ Clinical trials have largely ignored the underlying interindividual pathophysiological heterogeneity and have not used stratification for type of injury.^80^ Clinical studies in TBI are challenging because of the heterogeneity of the disease and lack of established surrogate endpoints to prove efficacy. Large animal models are uniquely able to capture the complex biological and physiological hurdles that are the major weaknesses to successful therapeutic development in clinical trials and may be used as a translational bridge to increase the likelihood of subsequent success in clinical trials.

We have demonstrated differences in the mitochondrial response after closed-head diffuse TBI and focal contusion TBI in porcine models.^55,81^ Preliminary PK analysis of a clinical study with NeuroSTAT reveals that the 20-mg/kg dose in piglets results in blood concentrations at steady state between the blood concentrations achieved in humans after treatment with 5 and 10 mg/kg/day (unpublished). Assuming that the trauma in the clinical study is similar to the trauma induced in piglets, and physiological differences between piglets and humans have a minimal impact on the brain concentrations of the drug, similar brain concentrations can be expected in humans.

A limitation of the study may be the therapeutic window in relation to the effect, with animals receiving cyclosporine 1 h post-TBI, and further studies may need to be completed to better define a therapeutic window for the NeuroSTAT formulation of cyclosporine. However, in our previous trials with the Kolliphor EL formulation in swine CCI injuries, we found positive outcome rates in our preliminary pre-clinical trials in animals treated 6 h post-CCI, leading us to conclude that there is likely a promising therapeutic window.^32^

We have demonstrated efficacy in a translational neuroimaging outcome metric in a large animal model of TBI, increasing the likelihood of a translation to success in subsequent clinical trials. In addition to the reduction in volume of injury, treatment improved cerebral metabolism of the pericontusional “at-risk” tissue measured at day 5 post-TBI consistent with cyclosporine's mechanism of action. NAA, one of the most abundant amino acids in the brain, is thought to be a marker of neuronal viability and mitochondrial health. Measuring NAA with ^1^H-MRS is a clinically valuable tool for patient prognosis and has been associated with patient outcomes, including Glasgow Outcome Scores.^82,83^ Altogether, cyclosporine as a treatment for TBI has a demonstrated track record unparalleled to other drug candidates in the pipeline. We believe our pig model is of high relevance for the predicted effects in humans, warranting and improving rapid translation to clinical trials. An open-label, nonrandomized phase II safety PK study of NeuroSTAT has recently been conducted in patients with severe closed TBI, and these data will lay the foundation for the next clinical trial focusing on efficacy.
